# Successful Aging and Happiness Level in the Elderly: The Mediating Role of General Self‐Efficacy

**DOI:** 10.1111/jocn.17711

**Published:** 2025-03-14

**Authors:** Özlem Özlü, Songül Duran

**Affiliations:** ^1^ Elderly Health Program, Institute of Health Sciences İzmir Demokrasi University İzmir Türkiye; ^2^ Health Services Vocational College İzmir Demokrasi University İzmir Türkiye

## Abstract

**Aim:**

This study aims to determine the relationship between successful aging and happiness levels in the elderly and to explore the mediating role of general self‐efficacy in this relationship.

**Design:**

This is a quantitative research study designed using a correlational research model between March and May 2022.

**Methods:**

The Mini‐Mental State Examination, the Descriptive Information Form, the Successful Aging Scale, the Oxford Happiness Questionnaire Short Form and the General Self‐Efficacy Scale were filled out by 144 elderly individuals living in a nursing home.

**Results:**

It was determined that the participants had high levels of successful aging behaviours/attitudes and good levels of happiness. It was determined that general self‐efficacy had a ‘partial mediating role’ in the relationship between successful aging and happiness.

**Conclusion:**

Due to the ‘partial mediating role’ of general self‐efficacy related to happiness and successful aging, increasing self‐efficacy may positively impact happiness and successful aging.

**Implications for the Profession and/or Patient Care:**

Healthcare professionals should prioritise practices and training that support the self‐efficacy of the elderly for their successful ageing and happiness. Within this scope, interventions can be made to increase the elderly's participation in self‐care, active engagement and intrinsic motivation.

**Impact:**

This study determined whether general self‐efficacy has an effect on successful aging and happiness in the elderly. It was determined that general self‐efficacy has a ‘partial mediating role’ in the relationship between successful aging and happiness. The general self‐efficacy of elderly individuals is an important issue to focus on. Successful aging includes functional ability, independence and quality of life aimed at maintaining health and well‐being in old age. Self‐efficacy, which is thought to have an effect on successful aging, is an important determinant in initiating and maintaining behaviours that improve the health of the elderly.

**Reporting Method:**

This study was reported adhering to the Strengthening the Reporting of Observational Studies in Epidemiology checklist.

**Patient or Public Contribution:**

The elderly individuals residing in a nursing home were included in the study. All participants were selected from a single nursing home. This nursing home accommodates both outpatient and bedridden elderly individuals. One section is reserved for individuals diagnosed with dementia.

**Summary**

- What does this paper contribute to the wider global clinical community?
  ○ Successful aging allows individuals to be physically, socially and mentally active and harmonious, and positively affects their happiness levels.
  ○ Self‐efficacy plays a role in increasing the happiness and general life satisfaction of older individuals by supporting both their independence and psychological resilience.
  ○ Training programmes can be organised to increase the self‐efficacy of the elderly living in nursing homes, which positively affects their successful aging and happiness.

## 1 Introduction

The combination of declining fertility rates and improving health conditions and longevity has led to an increasing number and proportion of elderly people worldwide (Ismail et al. 2021). This shift places significant pressure on social security systems, medical services and elderly care, while also requiring a re‐evaluation of strategies for achieving healthy aging (Zhou et al. 2025). The aging trend in society makes it particularly important to examine how successful aging can be achieved (Takács and Nyakas 2021). As the aging population increases, chronic diseases such as diabetes, high blood pressure and cognitive impairment may increase. One solution for countries to address this problem is to foster successful aging (Karamivand 2022). The term successful aging has been defined to include the absence of disease or disability, high cognitive and physical functioning, active participation in life, functional ability, independence and quality of life (Rodriquez 2020).

Literature suggests that successful aging requires indicators such as happiness, psychological well‐being, and effective adaptation, as well as the maintenance of physical and cognitive efficiency, the absence of disease and disability and continuous and active participation in social life (Çol et al. 2022). In addition, the inability to perform daily living activities due to age‐related physical stress and decreased organ functions negatively affects successful aging (Özsungur 2021). Maintaining physical, mental, spiritual, economic and social health can help individuals choose to age happier and avoid many of the problems associated with aging (Tiwery et al. 2023). Successful aging focuses on the importance of not only living longer but also living well (2024). Successful aging ensures that older adults remain active and productive members of society (Zábó et al. 2023). ‘Successful’ aging or the pursuit of happiness serves as a continuation of deeper human desires for meaning, well‐being and fulfilment throughout life.

## 2 Background

There are three basic criteria in the successful aging model developed by Rowe and Kahn. Accordingly, individuals must avoid disease and disability, ensure productivity and social participation in society and maintain cognitive and physical functions for successful aging (Rowe and Kahn 1997). This model has been criticised for overlooking important social and contextual factors and focusing solely on external and objective criteria (Kunuroglu and Vural Yuzbasi 2021). Another study defined successful aging as adapting behaviours to increase and/or optimise enjoyment and life satisfaction according to abilities, values and resources (Gonot‐Schoupinsky et al. 2022).

Successful aging means maintaining social circles and vibrant relationships while preparing for old age, taking preventive measures to minimise health problems, striving to improve memory and physical functions, and maintaining a positive orientation towards life (Shorey and Lopez 2021). According to Reynolds and colleagues, a broad definition of successful aging should have the following components: (a) subjective well‐being (the degree to which an individual perceives that current demands or challenges exceed his or her ability to cope) in which perceived stress levels are low; (b) development, including eudaimonic well‐being such as meaning in life and close social relationships; (c) posttraumatic growth; (d) sustained remission or recovery in individuals with severe mental disorders, typically characterised by functional independence with the absence or significant reduction of symptoms (Reynolds et al. 2022). Successful aging also includes the ability to cope with physical functioning challenges (Özsungur 2020). However, prolonged life span may increase dependence on others and reduce quality of life; therefore, successful aging is desirable (Ding et al. 2020). Life in a nursing home contradicts the ideal of successful aging in almost every way. It suggests dependency, inactivity, passivity and detachment (Balkin et al. 2023). Impaired functioning, reduced independence and multiple medical problems place older adults living in institutional care at risk for both poorer social support and less successful aging (Howie et al. 2014). But people's views about their capacity to succeed have a powerful influence on their actions (Yao 2019). Promoting successful aging improves the quality of life and well‐being of older people while reducing health care costs (Gu et al. 2023). Achieving successful aging is also influenced by an individual's lifestyle and health behaviours (Zhou et al. 2025). Successful aging is associated with high self‐efficacy (Teater and Chonody 2020). Self‐efficacy affects which behaviours a person chooses, how much effort he/she makes in the face of problems or different experiences, and how long he/she maintains this effort. For this reason, a high self‐efficacy perception increases one's efforts to struggle and cope (Bandura and Adams 1977). Self‐efficacy is a factor that increases self‐esteem and reduces anxiety levels (Won et al. 2019). Bandura emphasised four main sources of self‐efficacy. The first is mastery experiences in overcoming obstacles. Mastery experiences build coping skills and provide control over possible threats. The second is the variety of experiences provided by social models and seeing people similar to themselves who successfully perform similar behaviours. These experiences are considered the most effective source of efficacy. The third is their own belief that they have what it takes to succeed. The fourth is changing their negative emotions and misinterpreting their physiological state. Physiological state can affect the level of self‐efficacy when they interpret their somatic symptoms according to negative arousal. People who believe that they can manage these threats tend to be less disturbed by these threats (Shorey and Lopez 2021). Self‐efficacy refers to an individual's belief in their ability to execute courses of action required to produce specific outcomes and is directly related to health promotion and self‐care behaviours in the elderly (Ayhan 2023). Self‐efficacy is effective in happiness and psychological resilience levels (Arslan 2021). In their study on adult individuals, Ayyıldız and colleagues found that there was a positive relationship between self‐efficacy sub‐dimensions and happiness and emotion regulation (Ayyıldız and Sunay 2021). However, no study addressing this relationship among elderly people living in nursing homes has been found. Self‐efficacy is a mediator between knowledge and self‐care (Farley 2020). In a study conducted with older adults, life satisfaction was found to be higher in those who could do their daily tasks on their own and did not have sleep problems (Altay and Avcı 2009). In a study conducted with the elderly, it was seen that the factors affecting life satisfaction include the ability to perform daily living activities and social functionality (Khodabakhsh 2022). Implementing programs that will increase the self‐efficacy of individuals will positively affect successful aging and happiness.

It is thought that one of the factors that play a role in the successful aging and happiness of the elderly is self‐efficacy. This study aims to examine the relationship between successful aging and happiness levels in elderly individuals and to determine the mediating role of general self‐efficacy in this relationship. By focusing on the mediating role of general self‐efficacy, we aim to contribute to the development of effective strategies that promote successful aging and happiness among residents. For this purpose, a survey was applied to elderly individuals participating in the research in the nursing home where the research was conducted, and the following questions were sought:

1. Is there a significant relationship between successful aging and happiness in elderly individuals living in nursing homes?
2. Is there a significant relationship between successful aging and general self‐efficacy in elderly individuals living in nursing homes?
3. Is there a significant relationship between general self‐efficacy and happiness in elderly individuals living in nursing homes?
4. Is there a mediating role of general self‐efficacy in the relationship between successful aging and happiness in elderly individuals living in nursing homes?

## 3 Methods

### 3.1 Design

This study is a quantitative research design using the relational screening model. The STROBE checklist was followed to ensure the quality of the study (Cuschieri 2019). The relational screening model is a research method that aims to determine the relationships, interactions and correlations between variables.

### 3.2 Population and Setting

This study was conducted with 144 elderly individuals living in a nursing home. This nursing home is an institution affiliated with the municipality. Both bedridden and ambulatory elderly individuals receive services from this institution. It is one of the largest nursing homes in the city. This was chosen as the data collection location due to the ease of obtaining permits and transportation, and because it is an institution that houses elderly individuals heterogeneously.

### 3.3 Inclusion and/or Exclusion Criteria

Individuals who were 65 years of age or older, had no known dementia, scored 18 or higher on the Mini Mental State Examination (MMSE), and volunteered to participate in the study were included in the study. The Mini Mental State Examination (Folstein et al. 1975) was administered to the participants, and individuals who scored 18 points or above were included in the study. Individuals with inadequate visual acuity asked the researcher to read the question. The nursing home selected as the study area has a total capacity of 425 people, and 83 people were divided into geriatric units and Alzheimer‐dementia patient rooms, while the remaining 342 people were divided into healthy elderly. The survey was applied to the elderly individuals living in the nursing home with the deliberate sampling method until the sample was reached, and those who met the conditions were included.

### 3.4 Sample Size

The sample size for this study was determined using statistical power analysis methods, and the G*Power analysis program was used for sample size calculation (Faul et al. 2009). In our study, the effect size (Cohen *d*) was assumed to be at a hypothetical medium level (0.5), the margin of error (*α*) was accepted as 5%, and the power (1‐*β*) as 90%. Based on these parameters, the calculated sample size was determined to be 144 individuals.

### 3.5 Measurement

The data of the study were collected using the ‘Descriptive Information Form’, ‘Successful Aging Scale’, ‘Oxford Happiness Scale Short Form’ and ‘General Self‐Efficacy Scale’, which aim to obtain sociodemographic information.

### 3.6 Descriptive Information Form

This form consists of 11 questions in total, one open‐ended and 10 closed‐ended, including basic demographic information such as age, gender, education level, marital status, income level, status of having children, smoking habits, person capacity of the room in the nursing home, length of stay in the nursing home, frequency of going out and frequency of visitors.

### 3.7 Successful Aging Scale

The Successful Aging Scale, adapted into Turkish by Hazer and Özsungur (2018) (Hazer and Özsungur 2018), was developed by Reker (2009). In its original form, it consists of 3 sub‐dimensions and 14 questions regarding the healthy lifestyle of the elderly regarding successful aging, their efforts to overcome a problem, and their attachment to life (Hazer and Özsungur 2018). The Turkish adaptation of the scale consists of two subdimensions, ‘Healthy Lifestyle’ and ‘Coping with Problems’, and includes a total of 10 items. ll items on the scale are positive and use a 7‐point Likert type format. The lowest score that can be obtained from the scale is 10, and the highest score is 70. A high score obtained from the scale indicates that the participant's level of achieving successful aging is positive. In the validity and reliability study conducted by Hazer and Özsungur, Cronbach's Alpha value was found to be 0.85 (Hazer and Özsungur 2018), and in this study, the Cronbach's Alpha value was found to be 0.831.

### 3.8 The Oxford Happiness Questionnaire Short Form (OHQ‐SF)

The Oxford Happiness Scale (OHS), a psychometric scale used to measure happiness levels, was revised by Hills and Argyle (2002) to create the 8‐item Oxford Happiness Questionnaire Short Form (OHQ‐SF) (Hills and Argyle 2002). The scale used in this study is the version adapted into Turkish by Doğan and Çötok (2011). The Turkish‐adapted scale is a single‐dimension, 7‐item, 5‐point Likert scale. The minimum score that can be obtained from the scale is 7, and the maximum score is 35. Higher scores indicate higher levels of happiness in individuals. The internal consistency coefficient for OHQ‐SF reliability is 0.74, and the test–retest reliability coefficient is 0.85 (Doğan and Çötok 2011). In this study, the Cronbach Alpha value was found to be 0.717.

### 3.9 The General Self‐Efficacy Scale (GSES)

Another data collection tool used in the study is the General Self‐Efficacy Scale, which is used to assess individuals' self‐beliefs and their ability to cope with the difficulties they may encounter in daily life. This scale was originally developed by (Schwarzer and Jerusalem 1995) and its Turkish adaptation was made by Aypay (2010). The scale contains statements aimed at determining how effective and capable an individual feels in overcoming challenges encountered in daily life. The scale is a one‐dimensional, positive 10‐item 4‐point Likert type. The Cronbach Alpha coefficient of the version adapted to Turkish by Aypay was 0.83 (Aypay 2010), while the Cronbach Alpha value in this study was found to be 0.887.

### 3.10 Data Collection

The study was conducted between March and May 2022. Data were collected via a survey from participants aged 65 and over living in a nursing home. For participants with severe vision loss or who preferred to have the researcher read the survey, the researcher read the survey aloud and recorded the participants' responses as they responded verbally. The survey was conducted while wearing vision and hearing aids, if necessary. It took approximately 10 min to complete all scales.

### 3.11 Data Analysis

Data were analysed using quantitative analysis methods with the SPSS 27 software. Within the scope of quantitative analysis, descriptive statistics of participants and descriptive findings of scale scores were evaluated using descriptive statistics methods such as arithmetic mean, standard deviation and frequency analysis. The relationships between successful aging, happiness and general self‐efficacy levels were determined using Pearson correlation analysis. In order to determine whether general self‐efficacy plays a mediating role in the relationship between successful aging and happiness, Hayes (Process) Analysis (Hayes 2018) was conducted in accordance with the Baron and Kenny (1986) model. In addition, comparisons of participants' successful aging, general self‐efficacy and happiness levels according to various variables were carried out using independent groups (unrelated samples) *t*‐test, one‐way analysis of variance (ANOVA) and Hochberg GT2 multiple comparison (post hoc) tests. All of these analyses were conducted at a 95% confidence interval and a significance level of *p* < 0.05.

### 3.12 Ethical Considerations

The ethics committee approval for this research was obtained by the Ethics Committee of a university (Decision No: 2022/03‐06, Date: 23.02.2022). Throughout the research, all procedures were followed in accordance with the principles of the Helsinki Declaration, and the rights and privacy of participants were respected at every stage of the research process. Participants were informed about the purpose and methods of the study, and their participation was entirely voluntary. Additionally, the researcher took great care to ensure the confidentiality and anonymity of the information obtained from the participants. There were no names on the surveys. The data was stored by the researcher. All data was coded and entered into the SPSS program.

## 4 Results

### 4.1 Descriptive Statistics for Scale Scores

Table 1 presents the descriptive statistics obtained as a result of the evaluation of the scales used in the study. The participants' successful aging score average was determined as 57.58 ± 9.25 and it was observed that they had very successful aging behaviours/attitudes. In addition, very high averages were determined in the SAS sub‐dimensions of healthy lifestyle and coping with problems, similar to the overall average. The average score for participants' general self‐efficacy was determined as 31.35 ± 7.25 and it is possible to say that they have a good level of general self‐efficacy. Furthermore, the average score for the happiness scale among elderly individuals was determined as 26.12 ± 4.93, indicating that they generally possess a happiness level above average.

**TABLE 1:** Descriptive statistics for scale scores.

Puan	*n*	Min.	Maks.	$\underline{x}$	*s*
Successful aging	144	33.00	70.00	57.58	9.25
Healthy lifestyle	144	9.00	21.00	18.03	2.84
Coping with problems	144	17.00	49.00	39.54	7.38
General self‐efficacy	144	13.00	40.00	31.35	7.25
The Happiness Scale	144	15.00	35.00	26.12	4.93

### 4.2 Relationships Between Successful Aging, Happiness and General Self‐Efficacy Levels

Pearson correlation analysis was performed to determine the relationships between successful aging, happiness and general self‐efficacy levels among elderly individuals, and the findings are given in Table 2.

**TABLE 2:** Relationships between successful ageing, happiness and general self‐efficacy levels.

		Successful aging	Healthy lifestyle	Coping with problems	General self‐efficacy	The happiness scale
Successful aging	Pearson *r*	—				
*p*						
*n*		144				
Healthy Lifestyle	Pearson *r*	0.745**	—			
*p*		**< 0.000**				
*n*		144	144			
Coping with problems	Pearson *r*	0.967**	0.549**	—		
*p*		< 0.000	< 0.000			
*n*		144	144	144		
General Self‐efficacy	Pearson *r*	0.667**	0.469**	0.655**	—	
*p*		< 0.000	< 0.000	< 0.000		
*n*		144	144	144	144	
The Happiness Scale	Pearson *r*	0.536**	0.497**	0.481**	0.547**	—
*p*		< 0.000	< 0.000	< 0.000	< 0.000	
*n*		144	144	144	144	144

** *p* < 0.01.

The relationship between successful aging and happiness in elderly individuals was found to be moderately positive (*r* = 0.536) and significant (*p* < 0.00) (Table 2). In addition, in the sub‐dimensions of successful aging; the relationship between a healthy lifestyle and happiness was found to be moderately positive (*r* = 0.497) and significant (*p* < 0.00); the relationship between coping with problems and happiness was found to be moderately positive (*r* = 0.481) and significant (*p* < 0.00). Furthermore, the proportion of variance explained between successful aging and happiness was found to be 28.7% (*r* ^2^ = 0.287).

The relationship between successful aging and general self‐efficacy in elderly individuals was found to be moderately positive (*r* = 0.667) and significant (*p* < 0.00). In addition, in the sub‐dimensions of successful aging, the relationship between healthy lifestyle and general self‐efficacy was found to be moderately positive (*r* = 0.469) and significant (*p* < 0.00); the relationship between coping with problems and general self‐efficacy was found to be moderately positive (*r* = 0.655) and significant (*p* < 0.00).

The relationship between general self‐efficacy and happiness in older individuals was found to be moderately positive (*r* = 0.547) and significant (*p* < 0.00). In addition, the ratio of general self‐efficacy to happiness explaining each other was found to be 29.9% (*r* ^2^ = 0.299).

### 4.3 The Mediating Role of General Self‐Efficacy in the Relationship Between Successful Ageing and Happiness

Hayes (Process) Analysis (Hayes 2018) was conducted to determine whether general self‐efficacy has a mediating role in the relationship between successful aging and happiness, and the findings are given in Table 3.

**TABLE 3:** The moderating role of general self‐efficacy in the relationship between successful ageing and happiness.

Model	Independent variables	Dependent variables	*b*	SE	*β*	*t*	*p*	%95 (*b*)	
								Min.	Max.
(1) Total effects model	Successful aging	Happiness	0.286	0.038	0.536	7.569	< 0.000**	0.211	0.360
(2) Moderating model	Successful aging	General self‐efficacy	0.522	0.049	0.667	10.66	< 0.000**	0.426	0.619
	General self‐efficacy	Happiness	0.232	0.062	0.341	3.748	< 0.000**	0.110	0.354
	Successful aging	Happiness	0.165	0.048	0.309	3.396	0.001**	0.069	0.260

** *p* < 0.01.

In Table 3, as seen in Model 1 (total effect model), successful aging has a positive and significant effect on happiness before general self‐efficacy is included as a mediating variable (*p* < 0.00). The standardised relationship coefficient indicating the strength of the relationship between the two variables is *β* = 0.536. After general self‐efficacy is included as a mediating variable, as seen in the first row of model 2, the effect of successful aging on general self‐efficacy is found to be positive and significant (*p* < 0.00). The standardised correlation value showing the strength of the relationship between the two variables is *β* = 0.667.

In addition, as seen in the second row of model 2, the effect of general self‐efficacy on happiness was found to be positive and significant (*p* < 0.00). The standardised relationship value showing the strength of the relationship between the two variables is *β* = 0.341. Therefore, the condition for general self‐efficacy to serve as a mediating variable demonstrating a significant effect on happiness through its own influence has been met.

**FIGURE 1:**
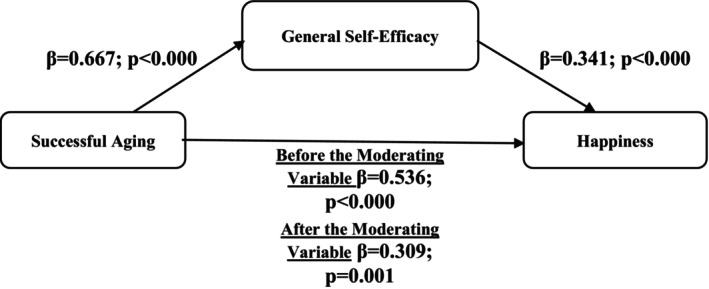
Findings regarding the moderating role of general self‐efficacy on the model.

However, for the mediating role to be established, the direct effect of successful aging on happiness must also become insignificant. When examined from this perspective, the direct effect of successful aging on happiness did not turn into insignificance, but was still found to be significant (*p* < 0.001). However, the strength of the relationship between the two variables decreased from *β* = 0.536 in model 1 to *β* = 0.309 in model 2. General self‐efficacy did not turn the direct relationship into insignificance, but weakened the strength of this relationship by 42.3% and ensured that it was transferred through itself. Therefore, it was decided that general self‐efficacy has a ‘partial mediating role’ in the relationship between successful aging and happiness (Figure 1).

## 5 Discussion

This research aimed to determine the levels of successful aging and happiness among elderly individuals, to examine the relationship between successful aging and happiness, and to identify the mediating role of general self‐efficacy in this relationship.

The successful aging score of elderly individuals was determined as 57.58 ± 9.25, and it was determined that they had successful aging behaviours/attitudes at a very successful level. There are similarities between the successful aging score average of our study and the data of the studies conducted in Brazil (Salamene et al. 2021) and Thailand (Anantanasuwong et al. 2022), and it can be said that despite the cultural differences between societies, similar positive aging dynamics are effective even in different cultures. In the literature, the successful aging score in studies conducted with elderly individuals in family health centres (Sert and Ilgaz 2024; Yalcinoz Baysal et al. 2020) and hospitals (Aydın and Aydın Sayılan 2022; Kütmeç Yılmaz 2020) is slightly lower than our score. While this difference can be explained by the fact that individuals living in a nursing home have stronger social support and a stable environment, the fact that individuals applying to the family health centre live in the community but with limited social support and environmental stability may have negatively affected the perception of successful aging.

In our study, when we analysed the happiness levels of the participants, it was found that the mean score of the OHQ‐SF was 26.12 ± 4.93 and this score corresponded to 68.3% when compared to the lowest and highest scores that could be obtained from the scale. The findings of this study are consistent with the happiness level obtained in our study (68.3%) and show that the participants generally have a happiness level slightly above the average of Türkiye. In a study conducted with elderly people living in nursing homes in Iran, the effect of music therapy on happiness was evaluated; it was observed that the happiness levels of elderly individuals increased significantly after the therapy, and it was determined that the therapy had a positive effect on happiness (Hazratian and Motaghi 2022). Increasing the number of practices that will positively affect the happiness level of the elderly (psychotherapy, music therapy, art therapy, etc.) may have a positive effect on the mental health of the elderly. It is recommended that these interventions be increased in nursing homes.

In this study, the general self‐efficacy score of elderly individuals was determined as 31.35 ± 7.25 and it was determined that they had a good level of general self‐efficacy. There are results consistent with these findings in the literature. In Tanrıverdi's study with COPD patients in Mardin, the GSES mean score was found to be 22.28 ± 5.86 (Tanrıverdi 2018). When compared with our study, it is seen that chronic diseases such as COPD negatively affect individuals' health perceptions and psychological resilience, whereas self‐efficacy levels may be higher in social and health‐supportive environments such as nursing homes. Similarly, in a study conducted in a nursing home in Iran, the participants' GSES mean score was found to be 22.95 ± 8.02, indicating a significant and positive relationship between general self‐efficacy and quality of life (Naseh and Heidari 2015). The GSES average score among young and old people aged 60–74 in rural areas in Shandong Province, China, was determined as 27.2 ± 8.1, and it was observed that the general self‐efficacy levels of elderly individuals were at a good level (Wang et al. 2019). These findings are consistent with the high general self‐efficacy levels in our study, revealing that the self‐efficacy levels of older individuals are generally at a positive level in different living conditions and cultural contexts. It is recommended that individuals be provided with training to support their general self‐efficacy and that motivational activities be carried out for individuals.

In the literature, a positive approach to life and happiness have been mentioned as components of successful aging (Badache 2023). In this study, the relationship between successful aging and happiness in older individuals was found to be moderately positive and significant. Similarly, studies conducted with older adults living in the community have found a statistically significant positive relationship between successful aging and the meaning of life (Kunuroglu and Vural Yuzbasi 2021; Salamene et al. 2021). A study conducted with women found that their ability to cope with life's challenges and maintain a positive perspective and perception positively influenced their successful aging (Özsungur 2020).

The relationship between successful aging and general self‐efficacy in elderly individuals was found to be moderately positive and significant. In a study conducted in Iran, the relationship of successful aging with lifestyle, spiritual intelligence and general self‐efficacy was examined in elderly individuals living in nursing homes, and a positive and significant relationship was found between general self‐efficacy and successful ageing (Mohammadi and Fardebrahimi 2023). Studies conducted with elderly people who do not live in nursing homes have also yielded similar results (Kim and Seo 2022; Torregrosa‐Ruiz et al. 2021).

In this research, it has been determined that general self‐efficacy has a “partial mediating role” in the relationship between successful aging and happiness. In another study, it was determined that daily activities significantly predicted the level of life satisfaction (Papi and Cheraghi 2021). In a study of older people, higher levels of general self‐efficacy provided a partial buffer against the negative impact of frailty on life satisfaction (Qin et al. 2020). A higher sense of self‐efficacy has been associated with better quality of life in psychological, social and environmental domains (Wojcieszek et al. 2023). Increases in self‐efficacy are linked to better health behaviours (such as exercise and diet) and reductions in the progression of multiple disease processes, including diabetes and cardiovascular disease (Hladek et al. 2020). High self‐efficacy has been found to have a positive effect on pain and physical activity in elderly patients with osteoarthritis (Degerstedt et al. 2020). Increasing self‐efficacy in elderly individuals with multiple chronic diseases can positively affect successful aging and happiness by increasing the ability to cope with diseases. In a study, it was determined that a life skills program can reduce the perception of aging and physical complaints during menopause and increase self‐efficacy in women (Dibaj et al. 2022). Healthcare professionals can develop nursing programs to maintain independence and increase self‐efficacy in older adults (Yong et al. 2021). Professionals should recommend interventions that focus on the capacities of older adults to maintain their health and lead a healthy lifestyle. Interventions should be designed to support autonomy and keep older adults socially and physically active (Bar‐Tur 2021). As emphasised in our study, the perception of self‐efficacy is a critical factor in this process, increasing both the happiness levels of individuals and their quality of life. These findings reveal the necessity of psychosocial interventions that strengthen the general self‐efficacy of individuals in order to support successful aging. A self‐efficacy development programme for older adults with mild cognitive impairment was found to effectively improve cognitive function, dementia knowledge, self‐efficacy and dementia preventive behaviours (Lee et al. 2023). Another way to increase self‐efficacy is through direct experience. Through live demonstrations or video tutorials, older adults can observe and imitate technical operations and the positive results that can be achieved. Additionally, providing a platform for older adults to learn from each other is vital (Fang et al. 2024). Verbal persuasion, another way to increase self‐efficacy, is to convey information, guidance, and advice so that an individual feels confident that he or she can accomplish a task (Juwita et al. 2023). In this sense, nurses can take part in persuading older individuals about their needs.

## 6 Limitations

The research was conducted in a nursing home, and since data were collected in a single institution, the results obtained from the research cannot be generalised globally/other nursing homes or elderly contexts without further investigation. In addition, the period in which the research was conducted, 2022, contains unique social and health dynamics due to the effects of the pandemic conditions. The effects of the COVID‐19 pandemic are among the factors that may affect the research results and may limit the validity of the results in a wider time period.

## 7 Conclusion

As a result of the research, it was determined that the elderly individuals who participated in this study had high levels of successful ageing and happiness. It was determined that there was a positive and significant relationship between successful ageing and happiness, and that general self‐efficacy had a mediating role in this relationship. This result shows us that increasing general self‐efficacy in older individuals may have positive results.

## Author Contributions

Özlem Özlü and Songül Duran contributed to the study concept and design, and interpreting the data, composed the statistical dataset, performed the analyses and wrote and revised the manuscript. All authors reviewed and approved the final version and no other person made a substantial contribution to the paper.

## Disclosure

The statistics were checked prior to submission by an expert statistician (https://www.tezyardimplatformu.com/).

## Conflicts of Interest

The authors declare no conflicts of interest.

## Data Availability

The data that support the findings of this study are available from the corresponding author upon reasonable request.
